# Incorporating a trauma‐informed perspective in HIV‐related research with transgender and gender diverse individuals

**DOI:** 10.1002/jia2.25976

**Published:** 2022-10-12

**Authors:** Susannah M. Allison, Karen L. Parker, Theresa E. Senn

**Affiliations:** ^1^ National Institute of Mental Health National Institutes of Health Rockville Maryland USA; ^2^ Sexual and Gender Minority Research Office National Institutes of Health Rockville Maryland USA

**Keywords:** transgender, gender diverse, HIV, trauma, violence, health behaviour

1

Transgender and gender diverse (TGD) individuals experience high rates of trauma and violence, including physical, sexual and psychological violence, as well as stigma and discrimination as a result of being a sexual and gender minority. These experiences may be exacerbated when intersecting, stigmatized identities such as some racial or ethnic identities are also present [1, 2, 3]. Thus, it is essential that HIV researchers understand how TGD participants’ experience of trauma impacts their research participation. A trauma‐informed lens, where everyone on the research team understands and recognizes potential signs and impact of trauma, where interactions are shaped by this recognition of trauma and where policies are developed to minimize trauma‐related distress [4], should be utilized throughout the HIV research process, including: developing research aims; staff training; study procedures; intervention development; and interpretation of findings (Figure 1). Utilizing a trauma‐informed lens in HIV research is critical regardless of the country or setting, given the universally high rates of transphobia and potential for related traumatic experiences. This article presents a set of suggestions for the field to consider when conducting HIV research with TGD individuals; these recommendations are not intended to set research or funding priorities.

**Figure 1:**
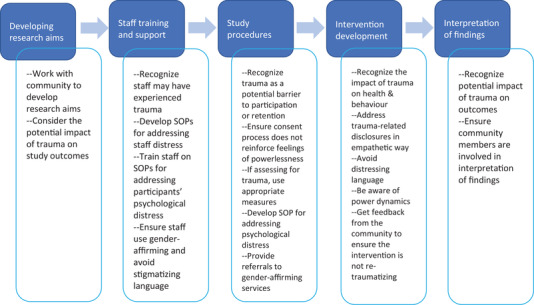
Incorporating a trauma‐informed perspective at each stage of the research process. Abbreviation: SPO, standard operating procedure.

Engagement with TGD community members should begin well in advance of any formal research development. Given the discrimination and trauma experienced by many TGD individuals, communities may not want to collaborate with researchers; establishing trust is critical, particularly when researchers are not from the TGD community. It is important to recognize that not all TGD individuals are active in TGD‐related organizations; additional outreach beyond these organizations is needed to incorporate a diversity of perspectives. Researchers engaging with TGD individuals should meet them in the communities where they live. TGD individuals who are advising the research team should be provided with the opportunity to talk about their and their community's health concerns and inform a collaborative approach to outlining the research goals. In addition, when developing research aims and questions, researchers should consider that trauma may influence the health outcomes of interest.

A trauma‐informed, trans‐sensitive environment is essential for staff and participants in any TGD‐related study. All staff should be trained in understanding trauma and its impacts [5]. It is important that staff avoid stigmatizing language [6] and use gender‐affirming language and materials during interactions with participants. While a research staff that reflects the study participants may increase participant comfort and trust and has been identified as important by TGD individuals [7], having a TGD research staff does not replace the need to incorporate a trauma‐informed perspective by everyone who interacts with study participants. Given that TGD individuals working on the study may themselves have histories of trauma, the research environment needs to support and empower all staff to address trauma and cope with secondary traumatic stress. Standard operating procedures, including appropriate referrals, need to be in place to address staff distress, re‐traumatization and potential burnout.

The potential impact of trauma should be considered throughout all study procedures. Many TGD individuals have experienced discrimination in healthcare or research settings; these prior experiences may be barriers to study participation. During the consent process, it is important to recognize the power differential between research staff and potential participants. Researchers should consider whether trauma assessments should be included in the assessment battery, as trauma may be an important predictor or moderator of outcomes. If traumatic experiences will be assessed, participants should be prepared for these questions through the informed consent process. In addition, researchers should recognize that trauma may take different forms depending on the participant population and should use appropriate measures to assess trauma. For example, measures of partner violence developed based on cisgender, heterosexual relationships may not adequately capture the range of partner violence experienced by TGD individuals (e.g. a partner threatening to “out” someone as transgender) [8]. Finally, researchers should develop standard operating procedures so that staff are consistently monitoring participants’ emotional reactions and are trained to handle psychological distress at any point. Specific plans should be developed to respond to instances of severe distress immediately by study staff, and referrals to gender‐affirming, accessible mental health services should be provided when needed. This may be a challenge in settings where there are few mental health and/or culturally competent providers; strategies used to deliver mental health services in low‐ and middle‐income countries, such as task sharing (i.e. when non‐specialists are trained and carefully supervised in the delivery of services) [9] and digital or mHealth interventions [10], may be useful tools in these settings.

In intervention‐focused HIV research, the potential impact of trauma on participation in and response to the intervention should be recognized. Researchers should understand that while behaviours addressed in the intervention may be harmful or unhealthy, they may also be an understandable reaction to the trauma participants have experienced [11]. Individuals may have experienced different types of traumas across social and structural levels and may cope in different ways [12]; interventions should acknowledge these different traumas and the multiple intersectional identities that may influence an individual's current behaviour and response to an intervention. Researchers should engage with the TGD community to ensure that the language and content of the intervention minimize distress. Those delivering the intervention should be aware of power differences due to roles and social identities and should empower participants to make informed decisions about their behaviours; telling participants what to do may reinforce participants’ feelings of powerlessness. If appropriate for the context and with trained facilitators, the intervention may directly address trauma. Even if the intervention does not directly address trauma, facilitators should be prepared for trauma‐related issues to arise and should respond in a non‐judgemental and empathetic way. If the intervention is delivered in a group format, hearing about others’ experiences may be upsetting for group members. Facilitators should be trained in how to balance sensitively acknowledging someone's experiences with the need to be sensitive to the potential distress of other participants. Discussing with the group up front how these situations will be handled may help to alleviate unnecessary challenges.

Finally, it is important to consider trauma when interpreting findings. Researchers should ensure that TGD community members are meaningfully involved in the interpretation and reporting of findings. Findings should not be over‐pathologized but should be interpreted through the lens that thoughts or behaviours may be an understandable response to traumatic experiences. If trauma was assessed in the study, it should be investigated as a potential influence on study outcomes.

Given the high rates of trauma and violence experienced by TGD individuals and the impact of trauma on health, HIV research that includes TGD individuals should employ a trauma‐informed approach across the entire research process. Further, other groups that are disproportionately burdened by HIV may also have high rates of diverse experiences with trauma, making a trauma‐informed approach potentially beneficial across all HIV research. Research teams should include those with expertise in trauma and sensitivity to the unique needs of TGD individuals, including those who are cognizant of the local and country context within which the research is being conducted. A trauma‐informed research approach should be adopted to address the significant HIV disparities among TGD individuals.

## COMPETING INTERESTS

The authors declare that they have no competing interests.

## AUTHORS’ CONTRIBUTIONS

SMA, KLP and TES contributed to the conceptualization of the manuscript, were involved in drafting the manuscript and critically revised the manuscript. All authors have read and approved the final manuscript.

## DISCLAIMER

The views expressed in this paper are those of the authors, and these views do not necessarily represent the official views of the National Institute of Mental Health, the Sexual and Gender Minority Research Office, the National Institutes of Health or the U.S. government.
